# Enhanced Electrical Properties of Atomic Layer Deposited La_x_Al_y_O Thin Films with Stress Relieved Preoxide Pretreatment

**DOI:** 10.3390/ma11091601

**Published:** 2018-09-03

**Authors:** Xing Wang, Hongxia Liu, Lu Zhao, Yongte Wang

**Affiliations:** Key Laboratory for Wide-Band Gap Semiconductor Materials and Devices of Education, School of Microelectronics, Xidian University, Xi’an 710071, China; xwangsme@xidian.edu.cn (X.W.); lzhaoxd@163.com (L.Z.); mikewyt@163.com (Y.W.)

**Keywords:** ALD, La_x_Al_y_O, SRPO, electrical properties

## Abstract

The impact of stress relieved preoxide (SRPO) interface engineering on the physical and electrical properties of La_x_Al_y_O films was investigated. It was proved that the SRPO pretreatment has little influence on the surface morphology of La_x_Al_y_O films and the chemical bond composition of La_x_Al_y_O/Si interface. However, the SRPO pretreated MIS capacitor displayed obvious improvement in decreasing the amount of trapped oxide charges and interfacial traps. As a result, a reduction of more than one order of magnitude in the gate leakage current density was obtained. The breakdown field strength and TDDB reliability of the La_x_Al_y_O film treated with SRPO were also enhanced.

## 1. Introduction

The aggressive scaling of complementary metal oxide semiconductor (CMOS) devices that came along with the scaling of SiO_2_ based gate dielectrics leads to unacceptable gate leakage currents [1]. To solve this problem, significant progress has been made on high-*k*/metal gate stack for the last two decades [2,3,4,5]. Presently, HfO_2_ in combination with metal gate electrodes have already been introduced for transistor production into the 45 nm Si-CMOS process line [6]. Nevertheless, in order to maintain the shrinking path, the development of higher permittivity gate dielectrics exceeding HfO_2_ is required for continued equivalent oxide thickness (EOT) scaling. Among various dielectric materials, La_x_Al_y_O is a promising alternative for the post-HfO_2_ films due to its high dielectric constant (*k*~25–27), wide band gap (>5 eV), and high band offsets with respect to Si (>2 eV) [7,8]. However, the deposition of high-*k* gate dielectric films, whether HfO_2_ or La_x_Al_y_O, on Si substrate, results in poorer interfaces between gate dielectrics and Si than that between thermally grown SiO_2_ and Si substrate [9,10]. High-density defects in the bulk of high-*k* gate dielectrics and in the interfaces of the gate stack structures are the major causes for instability problems as well as mobility degradation [11]. Therefore, the formation of some sort of high-quality interfacial layer seems desirable and unavoidable as a transition from high-*k* dielectric to Si substrate to reduce the density of interfacial defects [3]. Considering this, a defect passivation method of forming an extremely thin stress relieved thermal SiO_2_ interfacial layer by a stress relieved pre-oxide (SRPO) pretreatment [12,13] was carried out to improve the interfacial properties between La_x_Al_y_O dielectric and Si substrate in this paper. Attention was focused on the physical and electrical performance of La_x_Al_y_O film affected by this method.

## 2. Experimental

Two kinds of p-type Si (100) substrate surfaces treated with standard RCA cleaning process and SRPO pretreatment were prepared before the deposition of La_x_Al_y_O films. The SRPO pretreatment was carried out by placing the RCA cleaned Si wafer in O_2_ ambient at 1000 °C for 45 s to relieve interfacial stress [14], followed by dipping into a diluted HF solution (HF:H_2_O = 1:700) for 120 s to remove the thermally grown SiO_2_. La_x_Al_y_O films were grown in a Picosun R-150 atomic layer deposition reactor by alternately depositing La_2_O_3_ and Al_2_O_3_ using La(^i-^PrCp)_3_ and TMA as the La and Al precursor while O_3_ was used as the oxidant. A typical ALD growth cycle for La_2_O_3_ was 0.1 s La(^i-^PrCp)_3_ pulse/4 s N_2_ purge/0.3 s O_3_ pulse/10 s N_2_ purge, and an Al_2_O_3_ ALD cycle structure was composed of 0.1 s TMA pulse/3 s N_2_ purge/0.5 s O_3_ pulse/4 s N_2_ purge. By varying the number of ALD cycles, 5 nm La_x_Al_y_O films were deposited on these two kinds of Si substrate surfaces simultaneously at 300 °C. Post-deposition annealing (PDA) was carried out at 600 °C for 60 s in vacuum ambient.

The thickness of SiO_2_ on Si substrate before and after the SRPO pretreatment was measured by a spectroscopic ellipsometry (SE) system (J.A. Woollam Co. M2000U, Lincoln, NE, USA), as well as by the thickness of La_x_Al_y_O films before and after the PDA treatment. The surface morphology of the La_x_Al_y_O films was monitored using an atomic force microscope (AFM). X-ray photoelectron spectroscopy (XPS) was employed to investigate the bonding structures and chemical states of La_x_Al_y_O/Si interface. In order to evaluate the electrical properties of the La_x_Al_y_O films, MIS capacitors were fabricated by magnetron sputtering Ni/Al (20 nm/150 nm) electrodes on the surface of the wafers through a shadow mask (gate area of 7.07 × 10^−4^ cm^2^), and Al was sputtered as the back contact metal, followed by annealing in 97% N_2_/3% H_2_ ambient for 20 min at 400 °C. The electrical properties including capacitance-voltage (C-V), conductance-voltage (G-V), leakage current-voltage (I-V), and time-dependent dielectric breakdown (TDDB) characteristics were evaluated using an Agilent B1500A semiconductor parameter analyzer.

## 3. Results and Discussion

Compared with the standard RCA cleaning process, the steps of growing a relatively thick thermal oxide on the surface of Si substrate followed by dipping into a diluted HF solution were added in the SRPO pretreatment. The influence of these added steps on the surface morphology of Si substrates would certainly extend to the upper La_x_Al_y_O films grown later by ALD. Considering this, the SiO_2_ thickness on the surface of Si substrates was measured and analyzed statistically after different SRPO pretreatment steps. As shown in Table 1, the thickness of SiO_2_ was obtained by averaging the testing values of different positions on Si substrates, and the corresponding 95% confidence interval was also given. The thickness of native SiO_2_ on the surface of Si substrate was measured to be 2.31 nm. After the standard RCA cleaning process, the native SiO_2_ was reduced to 0.67 nm, with a narrow confidence interval of (0.596 nm, 0.744 nm), indicating the standard RCA cleaning process has a good effect in thinning the native SiO_2_ while keeping a high-quality surface uniformity.

When the thermal oxidation process was carried out to the RCA cleaning treated Si substrates, the thickness of SiO_2_ on the surface of Si substrates increased to 4.04 nm, which illustrates a relatively thick SiO_2_ layer was formed after the thermal oxidation process in O_2_ ambient for 45 s. Compared with the RCA cleaning treated Si substrate, the confidence interval appeared as a relatively wider distribution of (3.764 nm, 4.316 nm) at this time. Such a wider confidence interval indicates the thickness measurement results are relatively discrete, which may be caused by the inhomogeneous chemical reactions at different parts of Si substrate surface during the thermal oxidation process [15]. However, once the oxidized Si substrates were treated with diluted HF solution, the average thickness of SiO_2_ left on Si substrate surface turned out to be 0.65 nm, and the confidence interval was worked out as (0.537 nm, 0.763 nm). These values are comparable to what was observed after the standard RCA cleaning process, indicating the thermal oxidation process did not degrade the surface uniformity of Si substrate.

**Figure 1:**
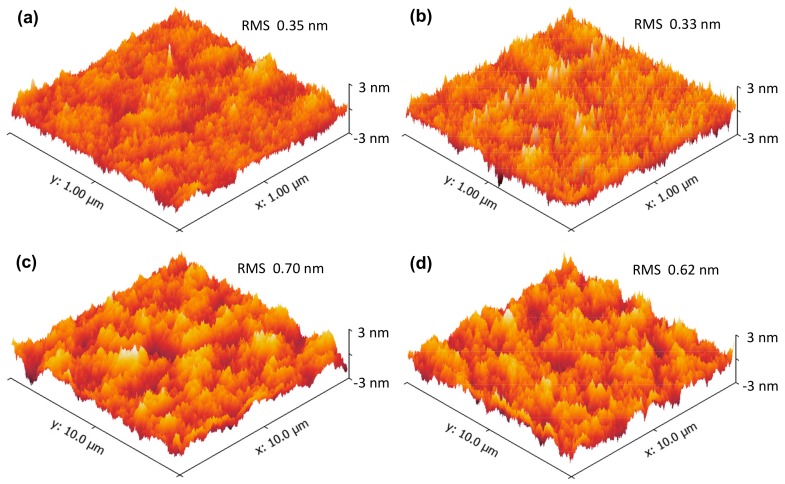
Atomic force microscope (AFM) images of La_x_Al_y_O films grown by ALD on Si substrates (**a**) without stress relieved preoxide (SRPO) pretreatment (1 × 1 μm^2^), (**b**) with SRPO pretreatment (1 × 1 μm^2^), (**c**) without SRPO pretreatment (10 × 10 μm^2^), and (**d**) with SRPO pretreatment (10 × 10 μm^2^).

For further investigating the influence of SRPO pretreatment on the surface morphologies of La_x_Al_y_O films, the surface of the La_x_Al_y_O films was examined by AFM. Figure 1 gives a typical three-dimensional AFM image of the annealed La_x_Al_y_O films. In a small scanning area (1 × 1 μm^2^), the surface roughness of the La_x_Al_y_O films without (Figure 1a) and with SRPO pretreatment, shown in Figure 1b, are 0.35 and 0.33 nm in root mean square (RMS), respectively. On the one hand, such small RMS values illustrate that the surface morphologies of La_x_Al_y_O films grown in this work are very flat and smooth, which is benefit from the nanoscale thickness controllability of the ALD technique [16]. On the other hand, compared with the RCA cleaning treated sample, there is no obvious change in the surface rough-ness of La_x_Al_y_O film with SRPO pretreatment, indicating the SRPO pretreatment has only a slight impact on the surface morphology of La_x_Al_y_O film. Even though scanned in a relatively large area (10 × 10 μm^2^), the surface morphologies of the La_x_Al_y_O films without and with SRPO pretreatment still show no obvious disparity in the surface roughness. That is, both of the two samples appear flat with uniform distribution, with the RMS value of 0.70, seen in Figure 1c, and and 0.62 nm, seen in Figure 1d.

**Figure 2:**
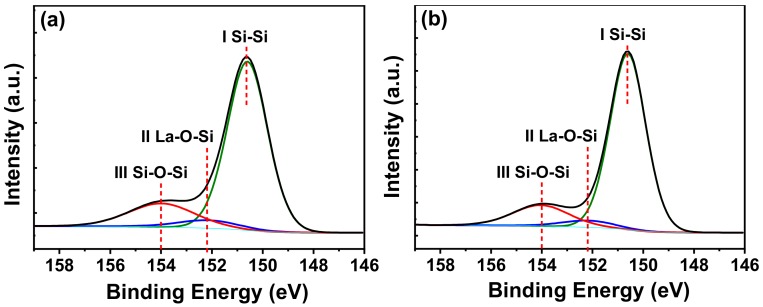
Shallow core-level spectra of Si 2*s* for the La_x_Al_y_O films grown by ALD on Si substrates (**a**) without stress relieved preoxide (SRPO) pretreatment, and (**b**) with SRPO pretreatment.

As shown in Figure 2, the variations in Si 2*s* XPS spectra for the annealed La_x_Al_y_O films were analyzed to investigate the chemical bonding states near the La_x_Al_y_O film and Si substrate interfaces. The La_x_Al_y_O films were sputter-etched with Ar^+^ ions for 15 s (0.26 nm/s) to obtain XPS signals from La_x_Al_y_O/Si interfaces. C 1*s* peak from adventitious carbon at 284.6 eV was used as a calibration reference during the XPS analysis [17]. The Si 2*s* spectra were fitted with three Gaussian-Lorentzian line shape peaks, which are at 150.6 (I), 152.2 (II), and 154.0 eV (III). Among the three peaks, peak I corresponds to the chemical bond of Si-Si, originating from Si substrate [18]. Peak II and III, corresponding to La-O-Si and Si-O-Si respectively, are likely present due to the existence of SiO_x_ and La-silicate which are the main components of interfacial layer (IL) between La_x_Al_y_O film and Si substrate [19]. For both La-O-Si and Si-O-Si peaks, the difference of the intensity between Figure 2a and Figure 2b is negligible, which indicates the SRPO pretreatment has almost no impact on the amount of La-O-Si and Si-O-Si chemical bonds. That is, the SRPO pretreatment on the Si substrate did not affect the chemical bond composition of IL in the later deposition and annealing process.

**Figure 3:**
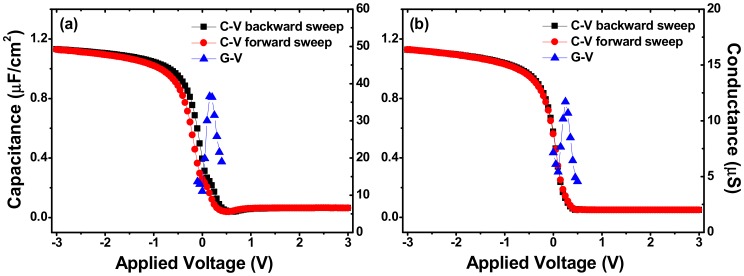
C-V and G-V characteristics measured at 100 kHz for the fabricated MIS capacitors using annealed La_x_Al_y_O films grown on Si substrates (**a**) without, and (**b**) with stress relieved preoxide (SRPO) pretreatment as insulators.

Figure 3 shows the C-V and G-V characteristics of the fabricated MIS capacitors using the annealed La_x_Al_y_O films as insulators. For simplicity, the MIS capacitors using La_x_Al_y_O films grown on Si substrates without and with SRPO pretreatment as insulators were assigned as capacitor S1 and capacitor S2, respectively. C-V measurements were performed at the frequency of 100 kHz. The gate applied voltage was biased from positive to negative (backward sweep) and followed by an opposite direction (forward sweep) to check the amount of C-V hysteresis. G-V curves were obtained simultaneously with the C-V curves swept from positive to negative. The flat band voltages (*V*_FB_) were extracted by fitting the C-V data using NCSU CVC program taking into account of quantum-mechanical effects [20]. The *V*_FB_ values for the backward swept C-V curves of capacitor S1, as seen in Figure 3a, and capacitor S2, shown in Figure 3b, were extracted as 0.005 and 0.142 V, respectively. However, the doping concentration of Si substrate used in this work is 5.0 × 10^15^ cm^−3^, considering the work function difference between Si substrate and Ni/Al metal gate electrode, the ideal *V*_FB_ could be worked out as 0.23 V [21,22]. So compared with ideal *V*_FB_, the C-V curves for both capacitor S1 and capacitor S2 show a *V*_FB_ shift towards the negative direction, which is an indication of the presence of effective positive oxide charges in the bulk of the insulator or in the interfacial region [23,24]. Taking ideal *V*_FB_ as a reference, a smaller shifting value of *V*_FB_ was obtained in capacitor S2 compared with that of capacitor S1, which indicates that, to a certain extent, the generation of effective positive oxide charges in the La_x_Al_y_O film and IL was restrained after the SRPO pretreatment on Si substrate.

Different degrees of counter-hysteresis were observed in the dual-swept C-V curves of Figure 3a,b. Such hysteretic behavior in the C-V characteristics is usually attributed to the trapping effects, or to be more specific, the counter-clockwise C-V hystereses indicate the existence of positive trapped charges in the interfacial region or in the bulk of the insulator [25]. The hysteresis width (Δ*V*_FB_) extracted from the dual-swept C-V curves for the MIS capacitor S1 and S2 are 0.131 and 0.015 V, respectively. For capacitor S1, a larger Δ*V*_FB_ of the dual-swept C-V curves illustrates the existence of more oxide trapped charges in the gate dielectric deposited on Si substrates without SRPO pretreatment. Assuming the two-dimensional distribution of traps in the vicinity of the interface contributing to the film capacitance, the oxide trapped charge density (*N*_ot_) for capacitor S1 and S2 can be estimated following the Equations [26,27]:

(1) $$C_{\mathrm{ox}\;}=C_{\mathrm{ac}}\left[1+\left(\frac{G_{\mathrm{ac}}}{\omega C_{\mathrm{ac}}}\right)\right]$$

(2) $$N_{\mathrm{ot}\;}=\frac{\Delta V_{\mathrm{FB}}C_{\mathrm{ox}}}{qA}$$

where *C*_ox_ is the gate insulator capacitance, *C*_ac_ is the measured accumulation capacitance, *G*_ac_ is the conductance in accumulation region, *q* is the electron charge (1.602 × 10^19^ C), *A* is the electrode area, and *ω* is the angular frequency. The calculation results are shown in Table 2. As expected, a remarkable decrease in *N*_ot_ was obtained for capacitor S2 (9.65 × 10^11^ cm^−2^) compared with that of capacitor S1 (1.06 × 10^11^ cm^−2^). It is generally known that trapped charge is one kind of oxide charges, so we can deduce that the positive trapped charges contribute to part of the effective positive oxide charges which cause the negative shift of *V*_FB_ as analysed before.

Besides, it is worth noting that, compared with what is shown in Figure 3b, the C-V curves for MIS capacitor S1, seen in Figure 3a) exhibit a more obvious anomalous hump phenomenon in the weak inversion region. It is reported that the appearance of humps in C-V curves is the characteristic features of the presence of interfacial slow traps [28]. Therefore, the more obvious anomalous hump phenomenon shown in Figure 3a indicates the formation of more interfacial traps at the La_x_Al_y_O/Si interface without SRPO pretreatment on Si substrate. The interfacial state density (*D*_it_) could be determined from the combination of the backward swept C-V and G-V characteristics using the following relation of Hill’s method [29]:

(3) $$D_{\mathrm{it}\;}=\frac{2}{qA}\frac{\frac{G_{\max}}{\omega }}{\left[{\left(\frac{G_{\max}}{\omega C_{\mathrm{ox}}}\right)}^{2}+{\left(1-\frac{C_{\max}}{C_{\mathrm{ox}}}\right)}^{2}\right]}$$

where *A* is the area of the gate electrode, *C*_ox_ is the gate oxide capacitance as defined in Equation (1), *G*_max_ is the peak value of conductance (obtained from G-V curves) and *C*_max_ is the capacitance corresponding to *G*_max_. The *D*_it_ value for capacitor S1 without SRPO pretreatment was worked out as 1.62 × 10^12^ eV^−1^ cm^−2^, and a much smaller *D*_it_ value of 4.19 × 10^11^ eV^−1^ cm^−2^ was achieved in capacitor S2 with SRPO pretreatment. Such a result is consistent with the varying degrees of humps in the C-V curves, indicating the SRPO pretreatment on Si substrate can effectively decrease the amount of interfacial traps.

**Figure 4:**
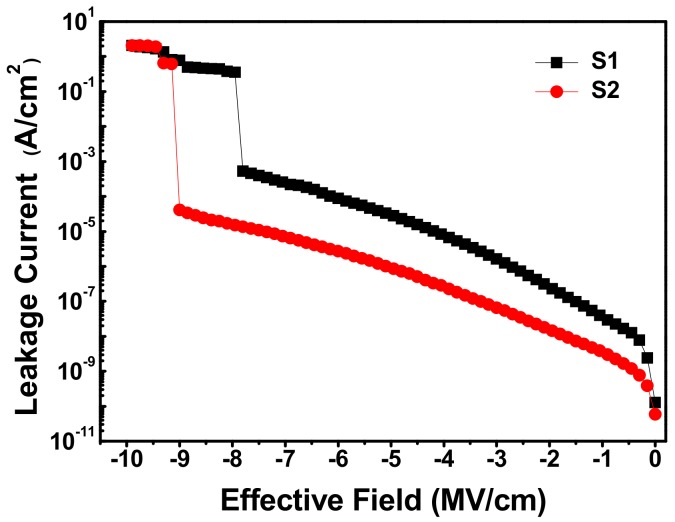
Leakage current-voltage characteristics for the fabricated MIS capacitors S1 and S2.

Figure 4 shows the leakage current density as a function of the applied electrical field for the fabricated MIS capacitor S1 and S2. At the applied electrical field of −7 MV/cm, the leakage current density for MIS capacitor S1 was measured to be 2.25 × 10^−4^ A/cm^2^. However, at the same applied electrical field, the leakage current density values for MIS capacitor S2 was determined as 7.24 × 10^−6^ A/cm^2^. Compared with MIS capacitor S1 without SRPO pretreatment, a decrease of more than one order of magnitude in the leakage current density was obtained for MIS capacitor S2 treated with SRPO process. Besides, it was found that the breakdown field strength for MIS capacitor S2 (~9.0 MV/cm) is appreciably higher than that of MIS capacitor S1 (~7.8 MV/cm). We ascribe this difference of gate leakage behaviors to the influence of La_x_Al_y_O/Si interface quality which was considered to be associated with structural defects [30]. The analyses on the C-V and G-V characteristics for MIS capacitor S1 and S2 revealed that the SRPO pretreatment on Si substrate contributes a favorable effect in restraining the generation of oxide charges and interfacial traps. Such a favorable effect is attributed to the reduction of structural defects nearby La_x_Al_y_O/Si interface such as oxygen vacancies and dangling bonds caused by the relief of interfacial stress, i.e., the re-combination and re-arrangement of interfacial chemical bonds, taking place in the thermal oxidation process (1000 °C, 45 s) of SRPO pretreatment. Less structural defects mean a smaller possibility to form a conduction path connecting Si substrate to the gate electrode in MIS capacitor S2 [31], resulting in lower leakage current density and higher breakdown field strength.

**Figure 5:**
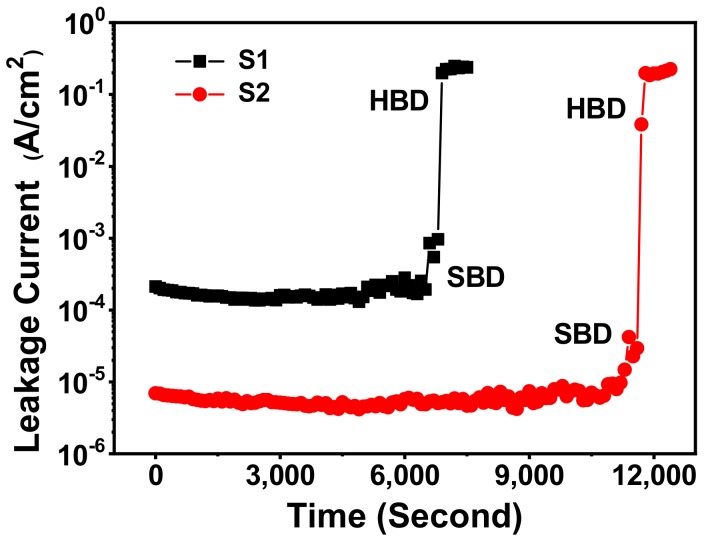
Time-dependent dielectric breakdown (TDDB) characteristics for the fabricated MIS capacitor S1 and S2 at the applied electrical field of −7.0 MV/cm.

Constant voltage stress (CVS) with a negative bias on the gate was applied to investigate the TDDB characteristics of the fabricated MIS capacitor S1 and S2 as shown in Figure 5. The applied electrical field was −7.0 MV/cm, which was close to the stress at which the hard breakdown (HBD) event occurred. It was observed that, with the increase of stress time, the TDDB characteristics for the two MIS capacitors changed in the same trend. That is, the leakage current decreases slightly in the early stage of the measurements by electron trapping phenomena, and then the current increases by hole trapping or by neutralization of trapped electrons to break down. According to the changing trend of gate leakage current, the TDDB characteristics could be defined into three regions, i.e., fresh region, soft breakdown (SBD) region and HBD region [32], respectively.

In the TDDB measurement, SBD has been reported to be closely dependent on the quality of interfacial layer [33]. Starting with the formation of multi unstable localized conduction paths in the interfacial layer, the gate stack does not breakdown completely in the SBD region. However, along with the increase of stress time, the localized conduction paths expand and drive into high-*k* layer, resulting in a gate punch through in the HBD region [34]. In this work, time-to-breakdown (*T*_bd_) is defined as the time at which HBD occurs. Then it could be observed that the *T*_bd_ for MIS capacitor S2 is about 11,700 s, which is much larger than that of MIS capacitor S1 (~6900 s). The extension in *T*_bd_ for MIS capacitor S2 was suspected to benefit from the improvement of La_x_Al_y_O/Si interface quality. On the one hand, compared with MIS capacitor S1 only treated with RCA cleaning, there should be fewer intrinsic defects nearby the La_x_Al_y_O/Si interface of MIS capacitor S2 treated with SRPO process; on the other hand, the chemical bonds of La_x_Al_y_O/Si interface formed in the thermal oxidation process of SRPO pretreatment should be more stable than those of RCA cleaning treated sample, which would decrease the probability of generating extra structural defects under the gate applied stress [35,36]. As a result, compared with MIS capacitor S1, a longer time, or a larger *T*_bd_, would be needed for accumulating defects to reach the critical point of HBD in MIS capacitor S2.

## 4. Conclusions

In summary, a comparative study on the physical and electrical properties of La_x_Al_y_O films grown by ALD on Si substrates treated with standard RCA cleaning process and SRPO pretreatment was provided. Some encouraging results were demonstrated with the SRPO pretreatment due to the reduction of bulk defects in La_x_Al_y_O film and the improvement of interface quality. Compared to the control sample, the MIS capacitor treated with SRPO process resulted in more than one order of magnitude gate leakage current density reduction, higher breakdown field strength and more stable TDDB reliability.

## Figures and Tables

**Table 1:** Thickness of SiO_2_ on the surface of Si substrates measured after different stress relieved pre-oxide (SRPO) pretreatment steps.

Process Step	Average Thickness (nm)	95% Confidence Interval (nm)
Pre-RCA cleaning	2.31	(2.211, 2.409)
Post-RCA cleaning	0.67	(0.596, 0.744)
Post-thermal oxidation	4.04	(3.764, 4.316)
Post-diluted HF solution dipping	0.65	(0.537, 0.763)

**Table 2:** Various parameters for the fabricated MIS capacitor S1 and S2.

Sample	*C*_ox_ (μF/cm^2^)	*V*_FB_ (V) Backward	Δ*V*_FB_ (V)	*N*_ot_ (cm^−2^)	*D*_it_ (eV^−1^ cm^−2^)
S1	1.18	0.005	0.131	9.65 × 10^11^	1.62 × 10^12^
S2	1.13	0.142	0.015	1.06 × 10^11^	4.19 × 10^11^
